# Loss of CDS1 impairs the tumorigenic characteristics of nasopharyngeal carcinoma by modulating lipid metabolism

**DOI:** 10.1080/19336918.2025.2520629

**Published:** 2025-06-25

**Authors:** Yifang Wang, Limei Li, Liudmila Matskova, Lixian Deng, Danping Li, Yi Huang, Haili Liang, Wen Wang, Ziyuan Liang, Jiaming Su, Weilin Zhao, Tingting Huang, Jiemei Chu, Zhe Zhang, Xue Xiao, Xiaoying Zhou

**Affiliations:** a Ministry of Education, Key Laboratory of High-Incidence-Tumor Prevention & Treatment (Guangxi Medical University), Nanning, China; b School Infirmary, Henan University of Urban Construction, Pingdingshan, China; c Institute of Molecular Biology and Biophysics, Federal Research Center of Fundamental and Translational Medicine (IMBB FRC FTM), Novosibirsk, Russia; d Institute of Molecular Biology and Biophysics, Federal Research Center of Fundamental and Translational Medicine, Karolinska Institutet, Stockholm, Sweden; e Department of Otolaryngology-Head and Neck Surgery, First Affiliated Hospital of Guangxi Medical University, Nanning, China; f Guangxi Zhuang Autonomous Region Institute of Product Quality Inspection, Nanning, China; g Life Science Institute, Guangxi Medical University, Nanning, China

**Keywords:** Nasopharyngeal carcinoma, CDP-diacylglycerol synthase 1, lipid droplets, NF-κB signaling pathway, inflammatory cytokines

## Abstract

The accumulation of lipid droplets (LDs) enhancing nasopharyngeal carcinoma (NPC) cell migration. We reveals that CDP-diacylglycerol synthase 1 (CDS1), an inhibitor of LDs formation, is significantly downregulated in NPC. Restoring CDS1 expression suppresses NPC cell growth, colony formation, tumorigenesis, migration, and invasion. The anti-cancer effect of CDS1 is attributed to its role in decreasing the intracellular LDs. Moreover, CDS1 promotes activation of the NF-κB signaling pathway, resulting in elevated levels of inflammatory cytokines within NPC cells. This is likely to enhance the immunogenicity of these cells, thereby reducing tumor volume in the in vivo model. These findings establish CDS1 as a novel suppressor of NPC by modulating LDs levels, suggesting potential therapeutic avenues aimed at limiting LDs accumulation.

## Introduction

Nasopharyngeal carcinoma (NPC) originates from the mucosal epithelium of the nasopharynx, it exhibits distinct ethnic and regional characteristics and is commonly found in southern China and Southeast Asia [1]. According to 2021 epidemiological data released by the World Agency for Research on Cancer, China represents 47.7% of the global NPC cases [2]. The etiology of NPC is multifactorial, involving Epstein-Barr virus (EBV) infection, particularly high-risk EBV variants, along with interactions between human and viral genetics, socio-regional variations, and environmental carcinogens [3]. Radiotherapy is highly effective for non-metastatic NPC patients, achieving a 5-year locoregional control rate exceeding 95%. However, prognosis remains poor for patients with distant metastasis [4]. Enhancing outcomes for NPC patients necessitates a deeper understanding of its pathogenesis and the discovery of effective therapeutic targets.

The Reprogramming of energy metabolism is a well-established characteristic of cancer [5], driven by cancer cells adapting lipid metabolism to meet their altered energy demands [6]. Lipid droplets (LDs), organelles comprising neutral lipids, serve as crucial intracellular energy reservoirs in the cytoplasm of eukaryotic cells [7]. Initially observed in mouse hepatoma cells in the 1950s [8], the presence of abundant LDs has since been linked to aggressive breast cancer variants like Paget’s disease [9], prompting investigations into lipid roles in tumorigenesis. LDs are now recognized in diverse cancers such as glioblastoma [10], cervical cancer [11], prostate cancer [12], renal clear cell carcinoma [13], cholangiocarcinoma [14], and pancreatic adenocarcinoma [15].

Recent studies underscore LDs’ role in enhancing tumor cell proliferation, resistance to apoptosis, and invasion, with LDs-rich tumors often exhibiting aggressive behavior and poor prognosis [16]. Active research focuses on understanding LDs functions across tumor types, including their synthesis, degradation mechanisms, and lipid uptake into cells. LDs also significantly influence cellular responses to inflammation, stress signals, and immune reactions against tumors. For instance, the upregulation of caveolae-associated protein 1 (CAVIN1) enhances lipid storage, activates inflammatory pathways, and promotes invasion in prostate cancer stromal cells [17]. Similarly, abnormal expression of fatty acid-binding proteins (FABP) in breast cancer triggers de novo fatty acid synthesis, NF-κB signaling activation, and inflammatory cytokine production [18].

Notably, NPC cells exhibit larger LDs compared to normal nasopharyngeal epithelial cells [19]. In NPC, the EBV-encoded latent membrane protein LMP2A augments LDs accumulation and enhances NPC cell metastatic potential by suppressing the expression of adipose triglyceride lipase (ATGL), a key lipid degradation enzyme [20]. This suggests impaired lipid degradation contributes to LDs accumulation in NPCs, yet the regulatory pathways and molecular details of LDs-associated inflammation in NPC remain unexplored. Further investigation is crucial to identify key molecules governing LDs synthesis pathways.

CDP-diacylglycerol synthase 1 (CDS1), pivotal in phospholipid metabolism by catalyzing CDP-diacylglycerol (CDP-DAG) from phosphatidic acid (PA), alongside with the synthesis of phosphatidylglycerol (PG), cardiolipin (CL) and phosphatidylinositol (PI) [21–23]. PA is able to synthesize diacylglycerol (DAG) and further to triacylglycerols (TAG), a key component of the lipid droplets. CDS1 suppresses LD expansion upon knockout in Hela cells and mouse fibroblasts [24,25]. In liver cancer, CDS1 downregulation due to promoter hypermethylation correlates with HBV infection, implicating its absence in HBV-positive liver cancer progression [26]. However, studies on CDS1 in various cancers, including NPC, remain limited.

This study specifically investigates the inactivation of CDS1 in NPC and demonstrates CDS1 re-expression effectively suppresses NPC cell proliferation, migration, and invasion. Furthermore, we elucidate how CDS1 reduces LDs accumulation in NPC cells, activating NF-κB signaling and downstream inflammatory factors, thus inhibiting NPC tumorigenesis.

## Material and methods

### Cell lines and tissue samples

The NP460 human immortalized epithelial cell line: Cultured in serum-free keratinocyte media (K-SFM) supplemented with growth factors (Gibco, Grand Island, NY, USA). NPC cell lines: C666–1 and HK1: Maintained in 1640 medium supplemented with 1% antibiotic and 15% fetal bovine serum (Invitrogen, Carlsbad, CA, USA). HONE1, 5-8F, and CNE1: Grown in high glucose DMEM medium (Gibco, Grand Island, NY, USA) containing 10% FBS and 1% antibiotic. All cells were incubated in a standard humidified carbon dioxide (CO₂) incubator.

All primary NPC samples and normal nasopharyngeal epithelium (NNE) control samples were sourced from the Department of Otolaryngology at the First Affiliated Hospital of Guangxi Medical University (Nanning, China). Written informed consent was secured from all participants. The 32 NPC samples, all newly diagnosed and untreated, were pathologically confirmed by seasoned pathologists based on WHO classification. For subsequent analysis, 15 NNE and 12 NPC samples were preserved in Trizol (Thermo Fisher Scientific) for RNA extraction, while 20 NNE and 20 NPC samples were treated with 10% paraformaldehyde and then embedded in paraffin.

### Real-time quantitative RT-PCR

Total RNA was isolated, and cDNA was synthesized according to previously established protocols [27]. The gene transcription was analyzed using SYBR Green Supermix by Qiagen, located in Hilden, Germany, on a QuantStudio 5 Flex Real-Time PCR System from Thermo Fisher Scientific, based in the USA. The relative expression levels of CDS1 mRNA were quantified and normalized against GAPDH mRNA. The results were computed using the 2-ΔΔCt approach. The specific primers ed were as follows:

CDS1-Forward:5’-GTGTTTGGATTCATTGCTGCCT-3’;

CDS1-Reverse:5’-TGGAAAGGGTACAAGCTCACT-3’;

GAPDH-Forward:5’-AAGCTCACTGGCATGGCCTT-3’;

GAPDH-Reverse:5’-CTCTCTTCCTCTTGTGCTCTTG-3’;

IL6-Forward:5’-GGTACATCCTCGACGGCATCT-3’;

IL6-Reverse:5’-GTGCCTCTTGCTGCTTTCAC-3’;

IL8-Forward:5’-ACTGAGAGTGATTGAGAGTGGAC-3’;

IL8-Reverse:5’-AACCCTCTGCACCCAGTTTTC-3’;

CXCL2-Forward:5’-CGCCCAAACCGAAGTCATAG-3’;

CXCL2-Reverse:5’-AGACAAGCTTTCTGCCCATTCT-3’;

IL1α-Forward:5’-GTTTAAGCCAATCCATCACTGATG-3’;

IL1α-Reverse:5’-GACCTAGGCTTGATGATTTCTTCCT-3’.

### Immunohistochemistry staining

Immunohistochemical (IHC) analyses were conducted following the protocol, as previously described [27]. Tissue sections of 3 μm thickness were dewaxed, hydrated, antigen-repaired, and endogenous peroxidase activity was blocked. The sections were incubated overnight with CDS1 mono antibody (dilution 1:30,34334, Novus, USA) at 4°C. Subsequently, this was followed by the application of a rabbit anti-mouse secondary antibody, which was incubated for 30 minutes at ambient temperature. Chromogenic detection was conducted using 3,3’-diaminobenzidine (DAB) (ZLI-9018, zsbb-bio, Beijing). Microscopic images were acquired with an Olympus C-5050 (Olympus, Japan). IHC scores, based on staining intensity and range of positive cells, were independently assessed by two investigators.

### Plasmids and transfection

Plasmid transfection into HONE1 and 5-8F cell lines was achieved using pCDH-CDS1 and control pCDH-entry vectors (Weizhen Biotechnology Co., Ltd., China). Transfections were performed with X-treme GENE HP DNA Transfection Reagent (Roche, Germany). Following transfection, cells were selected for stable integration by culturing in media containing 400 µg/ml G418 antibiotic (#G8160, Solarbio, Beijing, China) over a period of two weeks. The expression of CDS1 was verified via qRT-PCR and western blot assays.

### Cell proliferation assay

Cell proliferation influenced by CDS1 expression was assessed using a Cell Counting Kit-8 (Dojindo, Kumamoto, Japan). Cells were plated at 1.5 × 10^3^ cells per well in 96-well plates, with daily medium changes over 5 days. Proliferation was monitored by measuring the optical density at 450 nm every 24 hours, starting after an overnight attachment period. This assay was repeated five times to ensure reproducibility. Additionally, cells overexpressing CDS1 statically in 5-8F and HONE1 were treated with 30 µM oleic acid (#01001, Sigma Aldrich, USA) in their culture medium for 5 days, and cell proliferation was also assessed.

### Colony formation assay

The experimental and control cells were plated at 1,000 cells per well in 6-well plates and allowed to grow for two weeks, with the culture medium refreshed every third day. Following the culture period, the cell colonies were fixed, stained with crystal violet, and then imaged. Quantitative analysis of colonies was performed with the aid of Quantity One 1-D Analysis software, version 4.4.0.

### Wound healing assay

Experimental and control cells were inoculated in a 12-well plate of IBIDI cell plug-in (No. 80209, IBIDI, Germany) at a density of 3.5 × 10^4^ cells and cultured overnight in 10% DMEM medium to promote adhesion. After the cells formed a monolayer and reached confluence, the inserts were removed. Images were captured at 0 hours and every 6 hours using an inverted phase contrast microscope (TS100, Nikon, Japan). Wound closure was evaluated using ImageJ software. Each experiment was conducted in triplicate.

### Transwell assay

For the transwell assay, 2.5 × 10^4^ cells were seeded into the upper chamber of a 24-well Bio-Coat invasion chamber (BD, USA) that had been pre-coated with Matrigel. The lower chamber was filled with DMEM supplemented with 10% fetal bovine serum to act as a chemoattractant. After a 48-hour incubation period, non-invading cells were removed from the upper surface of the membrane using a cotton swab. Cells that had migrated to the lower membrane were fixed in 1% paraformaldehyde, stained with 0.1% crystal violet, and then photographed. In a related experiment, CDS1 overexpressing cell lines 5-8F and HONE1 were treated with 30 µM oleic acid (OA). Following treatment, these cells were evaluated using a similar transwell setup to assess their invasive properties under the influence of OA.

### In vivo tumorigenicity assay

Male BALB/c-nu nude mice, aged four weeks, were sourced from Guangxi Medical University Experimental Animal Center (China) for this experiment. A total of 12 mice were used, with six mice randomly assigned to each group. Each mouse was injected subcutaneously with 1.0 × 10^6^ Ctrl-5-8F cells in the right axillary region and an equal number of CDS1–5-8F cells in the left axillary region. All mice were included in the experimental results. The tumor growth was monitored bi-daily through two-dimensional measurements, and tumor volumes were calculated using the formula: volume (mm^3^) = length × width^2^  × 0.5. Tumor measurements were performed by an independent researcher who was blinded to the group assignments throughout the study to eliminate potential bias. Two weeks post-inoculation, the mice were euthanized by cervical dislocation method, and the tumors were excised and weighed. Confounding factors were not controlled for in this study. This study complied with the ARRIVE guidelines and adhered to the guidelines approved by the Animal Ethics Committee of Guangxi Medical University, and all procedures were conducted by approved protocols.

### Lipid droplet staining assay

The detection of neutral lipids in cells was carried out using BODIPY (493/503) fluorescent dyes (Invitrogen, USA). In brief, experimental and Ctrl cells (2 × 10^5^ cells) were cultured overnight in 12-well plates with cell crawl sheets. The cells were immobilized with a fixative for 30 minutes, followed by staining with a 1 μM BODIPY solution in the dark for an additional 30 minutes. Following this, the nuclei were stained using a DAPI solution (Solarbio, China) for visual observation. Confocal microscopy (FV3000, Olympus, Germany) was employed for observation and imaging.

### Triglyceride (TG) and total cholesterol (T-CHO) detection

Triglyceride (TG) and total cholesterol (T-CHO) levels were quantitatively assessed using specific assay kits (TG: A110–1–1, T-CHO: A111–1–1, Nanjing, China). Optical density (OD) readings at 510 nm were taken with a microplate reader (BioTek, Winooski, VT, USA).

### RNA sequencing and gene-set enrichment analysis

Total RNA quality was evaluated with an Agilent Bioanalyzer as per the manufacturer’s instructions. RNA sequencing was conducted using Illumina technology to create an RNA-Seq library via the Illumina Stranded mRNA Prep. The process involved mRNA isolation, cDNA synthesis, adapter ligation, library amplification, and indexing. Raw sequencing data were processed with Trimmomatic Manual V0.32 to remove low-quality reads, trim adapters, eliminate duplicates and obtain comprehensive transcript profiles. Differentially expressed genes (DEGs) between experimental and Ctrl cells were identified. Criteria for heterozygote screening included a fold change ≥ 1.2 and a *p*-value <.01.

### Western blotting

The nuclear proteins were extracted according to the instructions provided with the nuclear protein extraction kit (#R0050, Solarbio, China). Protein quantification followed the protocol outlined in the reference [28]. The detection utilized the following antibodies: CDS1 (1:800 dilution 28,338, SAB, USA), GAPDH (1:1000 dilution, HRP-60004, Proteintech, China), NF-κB p65 (1:1000 dilution, 8242S, CST, USA), and Histone 3 (D1H2, 1:1000 dilution, 4499, CST, USA).

### ELISA assay

ELISA kits (E-EL-H6156, E-EL-H6008, Elabscience, China) were utilized to quantitatively measure the levels of IL-6 and IL-8 proteins in experimental and Ctrl cells. Optical density (OD) readings at 450 nm were taken with a microplate reader (BioTek, Winooski, VT, USA).

### Statistical analysis

Data were analyzed using SPSS software version 26.0 (SPSS Inc., Chicago, USA). The differences between the two independent groups were assessed using the Student’s t-test. The p-values of *p < .05, **p < .01, and ***p < .001 were considered statistically significant when compared to the control group.

## Results

### CDS1 is significantly downregulated in NPC cells and tissues

**Figure 1.**
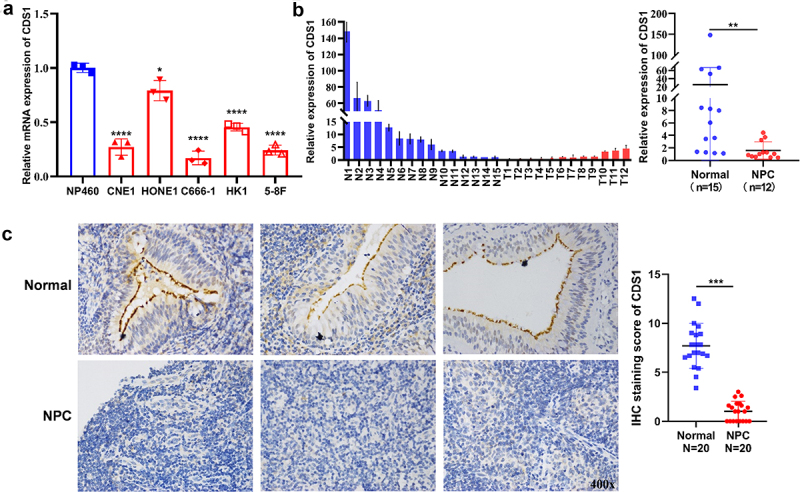
Down-regulation of CDS1 expression in NPC primary tissues and cell lines. (a) RT-PCR analysis of CDS1 transcription levels in normal nasopharyngeal epithelial cell line NP460 and NPC cell lines CNE1, HONE1, C666–1, HK1, and 5-8F. (b) RT-PCR analysis of CDS1 transcription levels in 12 NPC tissues and 15 normal nasopharyngeal epithelial tissues (NNE). (c) immunohistochemistry staining showing CDS1 protein expression in NPC tissues (*n* = 20) and NNE tissues (*n* = 20). (*p < .05, **p < .01, ***p < .001, ****p < .0001).

To investigate CDS1 gene expression in NPC, we conducted qRT-PCR analysis on the immortalized normal epithelial cell line NP460, and five tumor cell lines derived from primary NPC tumors (CNE1, HONE1, C666–1, HK1, and 5-8F). Figure 1 illustrates qRT-PCR results, revealing a remarkable decrease in CDS1 mRNA levels in NPC cells compared to NP460 cells (Figure 1a). Moreover, CDS1 mRNA was notably downregulated in NPC primary tissues (*n* = 12) compared to non-carcinoma nasopharyngeal epithelial (NNE) tissues (*n* = 15, Figure 1b). Immunostaining analysis further confirmed CDS1 protein expression in cytoplasmic regions of normal epithelial cells (NNE) (*n* = 20), contrasting with its absence in NPC cells (*n* = 20) (Figure 1c). These findings collectively indicate significant downregulation or absence of CDS1 expression at both mRNA and protein levels in NPC tumor cells.

### Restoration of CDS1 reduces LDs in NPC cell lines

**Figure 2.**
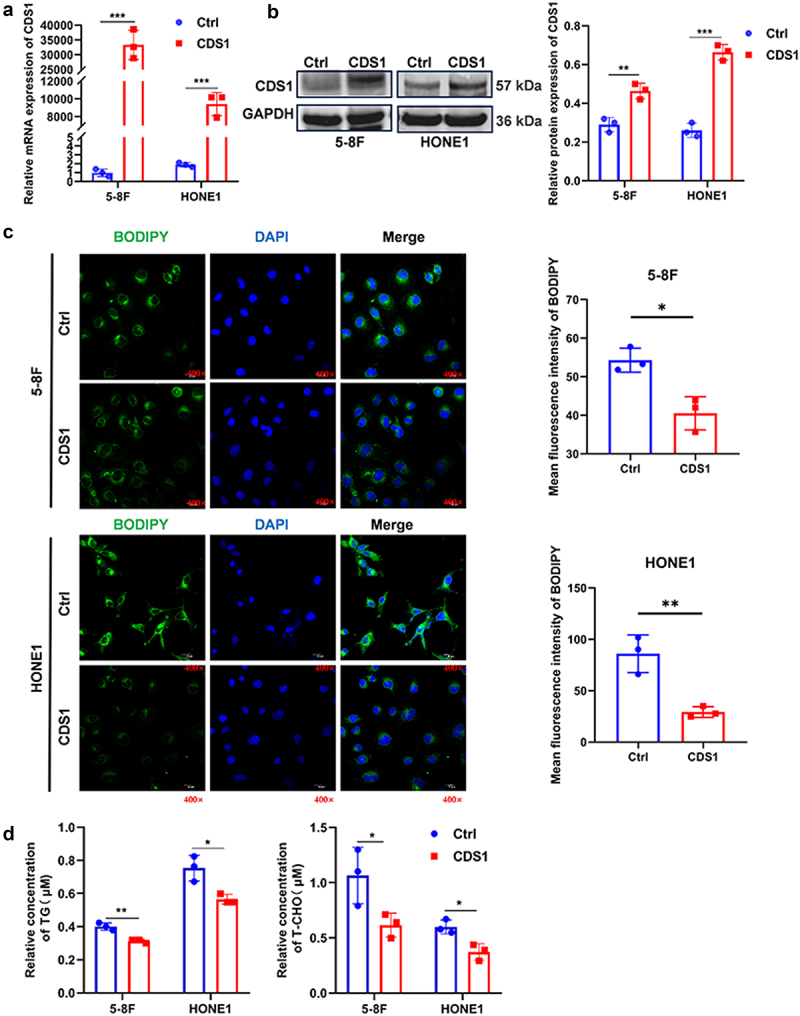
Overexpression of CDS1 reduces LDs content in NPC cell lines. (a-b) verification of CDS1 mRNA and protein expression by qRT-PCR and Western blot, respectively, following transfection with pCDH-CDS1 plasmid or empty vector (pCDH-entry) in 5-8F and HONE1 cell lines. (c) confocal microscopy images showing LDs stained with 1 μM BODIPY and nuclei with DAPI in NPC cells. (d) colorimetric assays measuring relative concentrations of intracellular triglycerides (TG) and cholesterol (CHO). Data represent mean±SD of three independent biological replicates. (*p < .05, **p < .01).

To investigate the functional role of CDS1 in NPC cells, we restored its expression levels in two NPC cell lines, HONE1 and 5-8F, generating stable cell lines designated as CDS1-HONE1 and CDS1–5-8F. Initially, we confirmed CDS1 overexpression in these cell lines using qRT-PCR and Western blotting assays (Figure 2a, b). Given CDS1’s critical involvement in lipid synthesis pathways [24,29], we examined its impact on lipid metabolism in NPC cells. For visualization of LDs formation, we employed the lipid-specific fluorescent dye BODIPY and conducted confocal microscopy analysis. We observed a significant reduction in LDs numbers in CDS1-transfected NPC cells compared to control cells (Figure 2c). Furthermore, to assess the inhibitory effect of CDS1 on LD formation, we conducted a quantitative analysis of the key components of LD: triglyceride (TG) and cholesterol (T-CHO). As anticipated, HONE1-CDS1 and 5-8F-CDS1 cells exhibited decreased levels of TG and T-CHO (Figure 2d). These findings strongly indicate that CDS1 acts as a negative regulator of LDs synthesis in NPC cells.

### CDS1 suppresses *in vitro* growth and *in vivo* tumorigenesis of NPC cells

**Figure 3.**
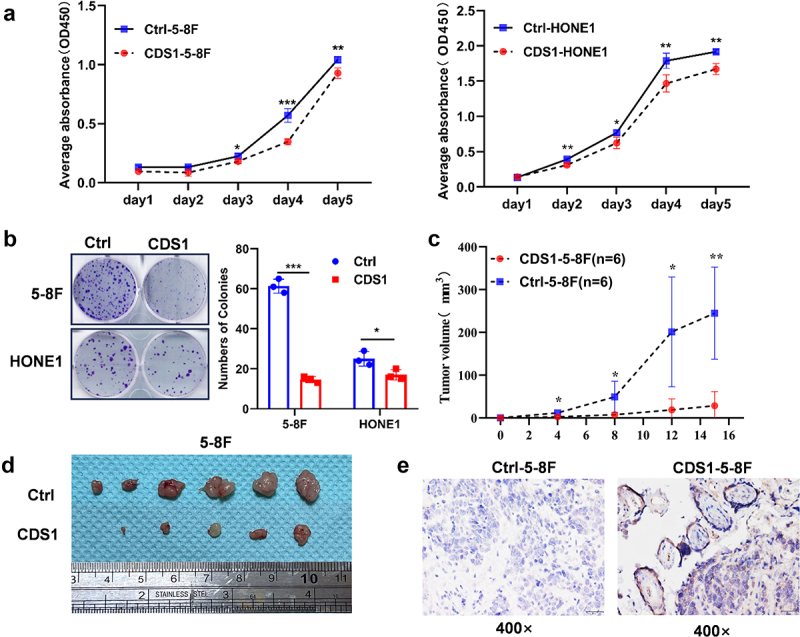
Overexpressed CDS1 inhibits the proliferation of NPC cells both *in vitro* and *in vivo*. (a) CCK8 assay assessing proliferation of 5-8F and HONE1 cells. (b) colony formation assay. (c) growth curve of CDS1–5-8F and pCDH-5-8F cells in nude mice. (d) xenografts from nude mice two weeks post-injection. (e) IHC staining was used to confirm the expression of CDS1 protein in xenografts. Data represent mean±SD of three independent biological replicates. (*p < .05, **p < .01, ***p < .001).

CCK-8 and colony formation assays were used to further investigate the impact of CDS1 on the malignant proliferation behavior of NPC cells. As shown in Figures 3a,b, overexpression of the CDS1 gene led to a significant reduction in both the proliferation and colony-forming ability of NPC cell lines 5-8F and HONE1. CDS1 not only inhibited the proliferation of NPC cells but this result was also confirmed through the establishment of the animal model in nude mice. Control cells exhibited a 100% incidence of tumors, whereas the CDS1 overexpressing 5-8F showed an 83% (5/6) incidence. Notably, the proliferating ratio of CDS1–5-8F cells in nude mice displayed a strikingly decreasing trend (Figure 3c). Evaluation of tumor size upon retrieval showed significantly smaller tumors induced by CDS1–5-8F cells compared to controls (Figure 3D). Immunohistochemistry analysis confirmed persistent CDS1 expression in xenografted 5-8F cells for over two weeks post-inoculation (Figure 3e). These results confirm that the overexpression of CDS1 can inhibit the growth of NPC cells both *in vitro* and *in vivo*.

### Restoration of CDS1 expression hinders migration and invasion of NPC cells

**Figure 4.**
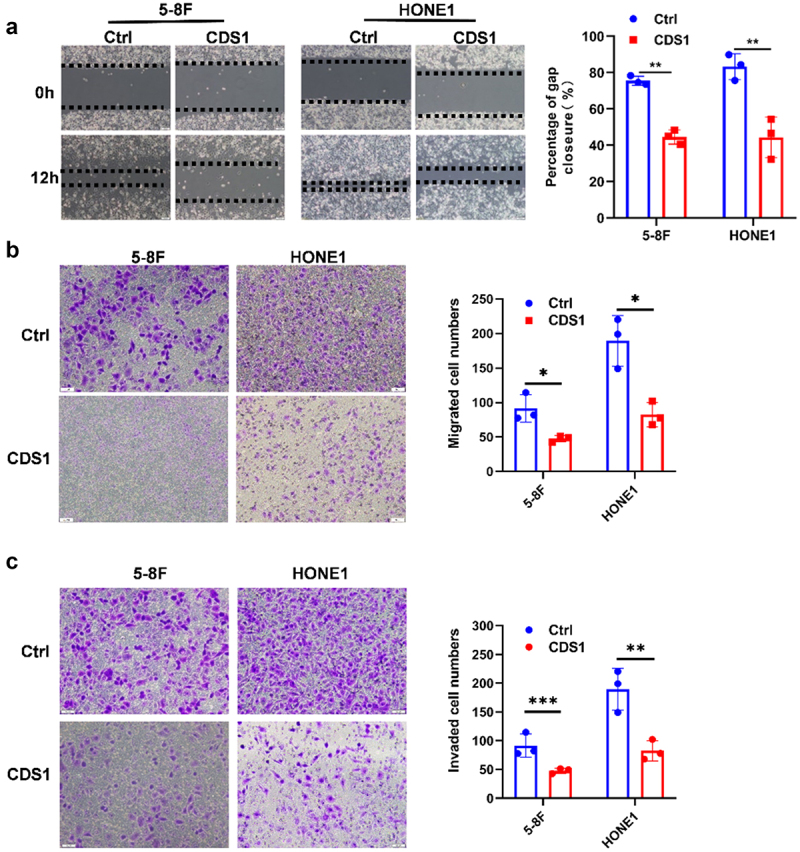
Increased CDS1 expression hinders the migration and invasion of NPC cells in vitro. (A) wound healing assay showing gap closure at 12 hr, quantified as percentage of original wound area using image J software. (B) Transwell assay without matrigel coating indicated that CDS1 inhibits the longitudinal migration ability of nasopharyngeal carcinoma cells. (C) Transwell assay demonstrating invasion of NPC cells, with violet-colored dots indicating invading cells stained with crystal violet. Data represent mean±SD of three independent biological replicates. (**p < .01, ***p < .001).

High invasiveness and migration ability are key characteristics of NPC. Here, we observed a significantly lower migration of the experimental cell lines to the wound area compared to the control cells. The gap closure area in CDS1–5-8F was 42%, whereas it was 78% in control cells. Similar results were obtained for HONE1-CDS1 cells (Figure 4a). Consistent with these findings, CDS1 overexpression was also demonstrated to significantly suppress the longitudinal migration capacity of nasopharyngeal carcinoma cells, further supporting its role in inhibiting tumor progression (Figure 4b). Additionally, the number of CDS1 overexpressed NPC cell lines penetrating the matrix glue was significantly lower than that of control cell lines, indicating that CDS1 inhibited the invasion of NPC cells (Figure 4c). These findings suggest that exogenous expression of CDS1 impedes the migration and invasion of NPC cells.

### Exogenous lipid supply reverses the inhibitory effect of CDS1 on the proliferation and invasion of NPC cell lines *in vitro*

**Figure 5.**
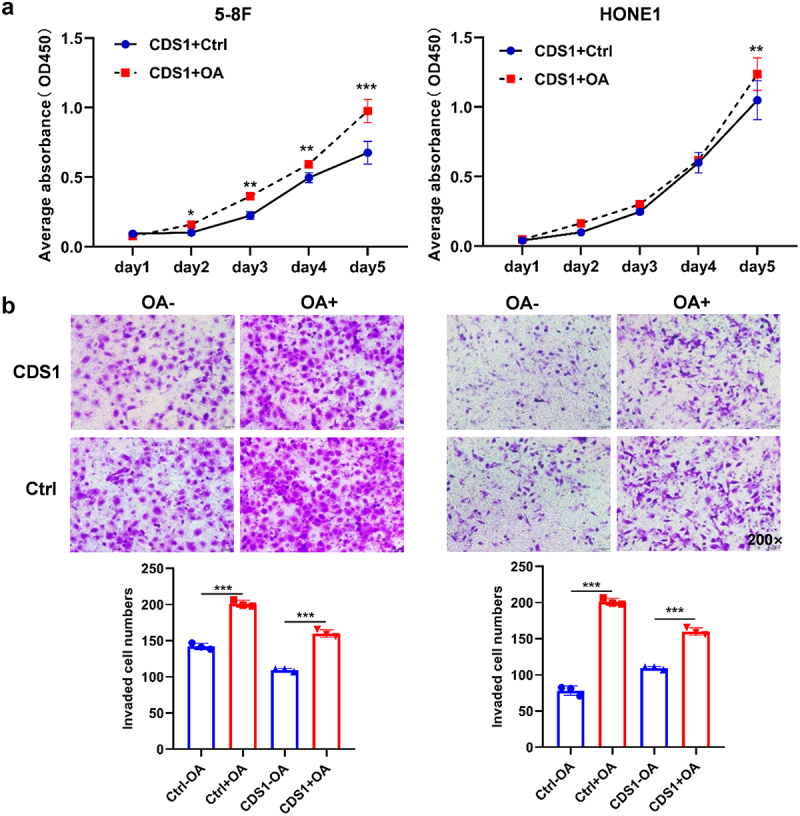
Exogenous lipid supply reverses the tumor suppressive effect of CDS1. (a) CCK8 assay confirms enhanced proliferation in CDS1-overexpressing NPC cells treated with oleic acid (OA). (b) Transwell assay. Data represent mean±SD of three independent biological replicates. Statistical significance denoted as *p < .05, **p < .01, ***p < .001.

Oleic acid (OA), a monounsaturated fatty acid prevalent in fat cells and blood, is known for its low toxicity compared to other fatty acids and serves as a substrate for LDs [30]. To investigate whether CDS1 affects the growth and metastasis of NPC cells by regulating intracellular lipids, a study was conducted using exogenous oleic acid (OA) to treat NPC cells that overexpress CDS1. As anticipated, the proliferation and invasion capabilities of NPC cells treated with OA were significantly augmented in comparison to the control group. This indicates that OA reversed the inhibitory impact of CDS1 on the proliferation and invasion of NPC cells (Figure 5a, b). This finding underscores the potential tumor-suppressive role of CDS1 through the modulation of lipid droplet levels.

### CDS1 activates inflammatory cytokine expression via NF-κB signaling pathway

**Figure 6.**
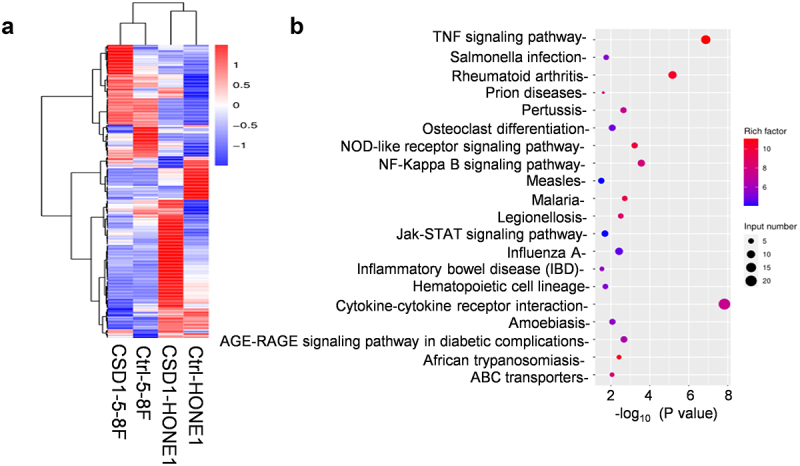
Analysis of transcriptional profiles altered by CDS1 overexpression using RNA-sequencing. (a) heatmap illustrating differentially expressed genes (DEGs) in NPC cell lines with and without CDS1.Overexpression (b) KEGG pathway analysis of DEGs these cell lines mentioned above.

To elucidate the molecular mechanism of CDS1 in NPC, we analyzed transcriptional profiles following CDS1 overexpression in NPC cell lines (Figure 6a). Our analysis revealed that upregulated genes induced by CDS1 primarily activate the TNF-α and NF-κB signaling pathways (Figure 6b). The nuclear transcription factor NF-κB plays a pivotal role in regulating cellular responses including inflammation, immune responses, apoptosis, and cell cycle regulation [31].

**Figure 7.**
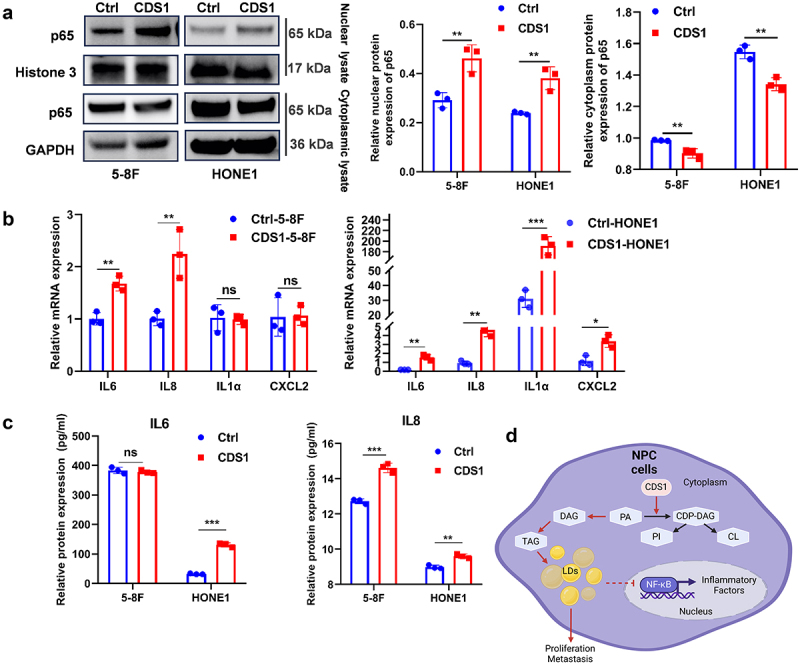
Activation of NF-κB (p65) signaling upon restoration of CDS1 expression in NPC cells. (A) Western blot analysis of nuclear p65 in indicated cell lines, with histone 3 as a loading control. (B) qRT-PCR quantification of inflammatory cytokine mRNA levels. (C) ELISA analysis of IL-6 and IL-8 cytokine protein levels. (D) diagram of CDS1 suppresses proliferation, metastasis, and inflammatory response of NPC cells through reducing LDs. Data represent mean±SD of three independent biological replicates. Statistical significance denoted as *p < .05, **p < .01, ***p < .001.

Notably, lipids have been shown to influence the transcriptional activity of NF-κB (p65) by affecting its nuclear translocation, as demonstrated in T cells [32]. In our previous study, we reported that lipids and inflammatory factors can impact NF-κB nuclear translocation in NPC cells [33]. Thus, we hypothesized that the observed increase in LDs levels in NPC cells may affect the nuclear transport of NF-κB and subsequently alter the transcriptional expression of downstream genes. To investigate this hypothesis about CDS1 expression in NPC cells, we examined the NF-κB (p65) protein expression in the nuclei of HONE1-CDS1, 5-8F-CDS1, and control NPC cells by extracting nuclear proteins. The results revealed upregulated NF-κB (p65) levels in the nuclei and decreased in the cytoplasm of NPC cells overexpressing CDS1, consistent with the NF-κB pathway activation as supported by RNA sequencing data (Figure 7a).

These findings were further validated by assessing the transcription of specific genes such as IL-6, IL-8, CXCL2, and IL-1α. Analysis showed significant upregulation of these genes in HONE1-CDS1 cells, with IL-6 and IL-8 significantly elevated in 5-8F-CDS1 NPC cells (Figure 7b). Protein analysis confirmed significant increases in IL-6 and IL-8 levels in HONE1-CDS1 cells, while IL-8 was notably elevated in 5-8F-CDS1 cells (Figure 7c).

Based on these findings, we have proposed a hypothetical model in which CDS1 catalyzes the conversion of phosphatidic acid (PA) to CDP-diacylglycerol (CDP-DAG). The subsequent decrease in triacylglycerol (TAG) accumulation affects the formation of lipid droplets (LDs). The reduction in LD levels leads to nuclear translocation of the NF-κB, which subsequently activates inflammatory transcription factors in NPC cells (Figure 7d).

## Discussion

Abnormal lipid metabolism is recognized as a contributing factor in NPC carcinogenesis. As molecular insights into metabolic alterations in NPC cells continue to emerge, mounting evidence supports the association between lipid metabolism changes and NPC development. A recent lipidomics study highlighted significant alterations in lipid components including phosphatidylinositols, free fatty acids, and diacylglycerols in the plasma of NPC patients in contrast with healthy donors [34]. Quantitative lipid analysis has shown potential for assessing distant metastasis and clinical outcomes in NPC [35]. Additionally, EBV infection, a major etiological factor in NPC, is associated with distinct lipid profiles. EBV, displaying latency type II, predominantly expresses viral messages including EBERs, BARTs, EBNA1, LMP1, and LMP2 [36]. EBERs, for instance, strengthen the translation of fatty acid synthase (FASN) in NPC [37]. Similarly, LMP1 induces FASN transcription mediated by sterol regulatory element-binding protein 1 (SREBP1) [38], while LMP2A inhibits the key lipid catabolic enzyme ATGL, potentially enhancing lipid accumulation in NPC cells [20]. These findings underscore EBV’s potential role in NPC carcinogenesis through lipid metabolism modulation.

Additionally, epigenetic modifications in NPC genomes contribute to abnormal expression of lipid metabolism-related molecules. Our previous study demonstrated that hypermethylation of the UbcH8 gene promoter in NPC cells results in its silencing, thereby compromising the function of the ISG15-conjugating enzyme UbcH8 and increasing intracellular LDs levels [19]. Reduced expression of miR-433-3p enhances an enzyme that is responsible for oleic acid synthesis, stearoyl-CoA desaturase 1 (SCD1), which promotes NPC progression [39]. Elevating long noncoding RNA (lncRNA) TINCR in NPC leads to cytoplasmic acetyl-CoA accumulation and increased de novo lipid synthesis [40]. Methyltransferase-like 14 (METTL14), the key writers of m6A modification, upregulates the expression of pinpointed ankyrin repeat domain 22 (ANKRD22) and may promote lipid metabolism reprogramming in NPC cells by interacting with SLC25A1 on mitochondria, thereby supporting the growth of NPC [41] In our study, we observed a significant downregulation of CDS1 in NPC. We demonstrated that restoration of CDS1 expression disturbs the growth of LDs in NPC cells. Although our examination of CpG island methylation in the CDS1 promoter region did not reveal significant differences between normal epithelial and NPC cells (data not shown), the mechanisms underlying CDS1 dysregulation warrant further investigation. Moreover, exploring the potential role of CDS1 activity in EBV entry into epithelial cells would be intriguing.

Functional analysis revealed that lipid accumulation in NPC cells, upon restoration of CDS1 expression, attenuates cell proliferation and migration, consistent with recent reports linking lipid metabolism to NPC pathogenesis. Lipid metabolism not only promotes the growth and motility, but also participates in other malignant behaviors. The identification of protein C receptor (PROCR) in NPCs underscores its role in supporting stemness through the regulation of lipid metabolism and mitochondrial dynamics [42]. In line with this, silencing leptin, as a regulator of energy homeostasis, attenuated TAG and cholesterol levels, thereby reducing the survival of NPC cells [43]. Targeting lipid biosynthesis and its utilization also serves as an effective adjuvant therapy for radiotherapy and chemotherapy. For instance, interference with FASN enhances the efficacy of radiotherapy for NPC [44]. Inhibiting the expression hypoxia-inducible lipid droplet-associated protein (HILPDA) leads to a reduction in LDs formation and a decrease in the mitochondrial cardiolipin level. Consequently, this process enhances the radiosensitivity of NPC cells by inhibiting mitophagy [45]. The utilization of fatty acids and maintenance of energy homeostasis occurs through β-oxidation of fatty acids (FAO), which produces more ATP than oxidation of carbohydrates [46]. Cancer cells may profit from this process. Indeed, active fatty acid trafficking and FAO was found in radiation-resistant NPC cells [47]. In support of this, interference with lipid synthesis has been shown to sensitize the chemoresistant NPC cells [40]. In other types of tumors, LDs accumulation also plays a pro-carcinogenic role. Particularly in clear cell renal cell carcinoma, the accumulation of large amounts of lipids is a prominent pathological feature. Multiple molecules and signaling pathways are involved in the regulation of LDs synthesis and FAO, promoting its malignant biological behavior [48,49]. As a tissue with abundant fat, increased intracellular triglyceride storage is a unique metabolic feature of ER+ breast cancer that contributes to endocrine therapy resistance [50]. Currently, various gene regulatory methods and targeted drugs that inhibit LDs accumulation are being developed [51,52], which undoubtedly highlights the significant role of LDs in tumors.

Numerous studies have implicated LDs in the regulation of cellular signaling pathways. To further elucidate the impact of CDS1 on modulating signal transduction, we analyzed the transcriptional profiles of CDS1-overexpressing NPC cells and control cells. KEGG pathway analysis of differentially expressed genes highlighted the NF-κB signaling pathway as prominently affected by CDS1. The NF-κB pathway governs inflammation, immune response, and cancer progression. Our previous findings indicated that LDs in NPC cells interfere with inflammatory response induced by bacterial cell wall components, partly by inhibiting NF-κB(p65) subunit translocation from the cytoplasm to the nucleus [53]. Therefore, we believe that LDs accumulation hampers the NF-κB nuclear translocation by physically trapping it. Here, we demonstrated that restoring CDS1 expression in NPC cells significantly increases nuclear NF-κB(p65) levels, leading to upregulation of downstream targets such as inflammatory cytokines IL-6, IL-8, and others. Thus, LDs accumulation likely hampers NF-κB-mediated inflammatory responses in NPC cells. Similarly, silencing fatty acid-binding protein 5 (FABP5) in increases LDs levels in cancer cell lines, inhibiting NF-κB activation [18]. Conversely, interfering with enzymes involved in triglyceride synthesis suppresses immune response-related gene expression, including NF-κB, IL-1β, and TNF-α [54]. In a type 2 diabetes rat model, reducing LDs size correlates with inhibition of the NF-κB pathway [55]. These findings underscore the context-dependent relationship between lipid metabolism and inflammatory signaling. While the present study highlights the potential of NF-κB pathway activation and subsequent cytokine production to enhance tumor immunogenicity, it is crucial to acknowledge the context-dependent and often dual nature of NF-κB signaling in cancer. Chronic or dysregulated NF-κB activation can exert potent protumoral effects in certain settings. The therapeutic strategies aimed at exploiting NF-κB for immunogenicity enhancement must carefully consider this delicate balance to avoid unintended promotion of tumor progression or immunosuppression.

In summary, our findings indicated that the downregulation of CDS1 in NPC contributes to LDs accumulation. By modulating lipid metabolism, CDS1 serves as a tumor suppressor, inhibiting NPC cell proliferation and metastasis. Additionally, CDS1 overexpression reduces LDs level, which in turn activates NF-κB-mediated inflammatory cytokines expression, potentially through NF-κB nuclear translocation. This mechanism may enhance anti-tumor immune response, leading to reduced tumor growth *in vivo*. Our findings underscore the pivotal role of CDS1 in NPC and propose that targeting lipid metabolism may represent a promising therapeutic approach for the management of NPC. However, further clinical validation is required to ensure its safety before it can be applied in practical therapeutic settings.

## Data Availability

The datasets generated during and analyzed during the current study are available from the corresponding author on reasonable request a data generated or analyzed during this study are included in this published article and its supplementary information files. The relevant data used in this study were obtained from the GEO and database (https://www.ncbi.nlm.nih.gov/bioproject/PRJNA1110849).
